# Evolution‐guided yeast complementation reveals functional differences in human *PSPH* variants

**DOI:** 10.1002/2211-5463.70308

**Published:** 2026-08-05

**Authors:** Mauricio Campa‐Álvarez, Diana Ascencio, Miguel Vallebueno‐Estrada, Eduardo González‐Orozco, Christian Eduardo Martínez‐Guerrero, Rafael Montiel, Alexander DeLuna

**Affiliations:** ^1^ Unidad de Genómica Avanzada Cinvestav Irapuato Mexico; ^2^ Centro de Investigación sobre el Envejecimiento Cinvestav Mexico City Mexico; ^3^ Departamento de Ingeniería Genética, Unidad Irapuato Cinvestav Mexico; ^4^ Unitat d'Antropologia Biològica, Departament de Biologia Animal, de Biologia Vegetal i d'Ecologia Universitat Autònoma de Barcelona Spain

**Keywords:** ancient DNA, heterologous complementation, phosphoserine phosphatase, serine biosynthesis, yeast functional assays

## Abstract

Deciphering how human genetic variants affect conserved metabolic enzymes is essential for understanding their evolutionary and clinical significance. Here, we combine sequence analyses of temporally stratified human genomes with a quantitative *Saccharomyces cerevisiae* complementation assay in a strain lacking *SER2*, the yeast gene required for the final step of L‐serine biosynthesis, to examine functional differences among human phosphoserine phosphatase (PSPH) variants. Population‐genomic comparisons between ancient hunter‐gatherers and present‐day humans identified two *PSPH* exons with elevated differences in nucleotide diversity, guiding the selection of two ancient‐genome‐prioritized variants (R27S and Q83H) for functional testing. To place their effects in functional context, we expressed each variant individually and compared complementation with the modern *PSPH* allele and two disease‐associated alleles (D32N and A35T) across multiple environmental conditions. Human *PSPH* enhanced growth of the *SER2* deletion mutant and revealed reproducible quantitative differences among alleles. The modern allele generally conferred the strongest complementation, while the ancient genome variants supported measurable but more condition‐dependent rescue, and the disease‐associated alleles showed the weakest complementation. These differences were broadly consistent across conditions, while specific environmental perturbations revealed context‐dependent shifts in effect size. Together, our results establish a scalable framework that links evolutionary genomics with experimental functional assays to identify and evaluate human metabolic enzyme variants with measurable *in vivo* effects.

## Graphical abstract

Ancient genomes can help guide which human genetic variants are tested experimentally. This study applies that idea to *PSPH*, a gene involved in serine biosynthesis, and uses high‐throughput yeast complementation to compare variant function. The findings reveal measurable differences among selected alleles and illustrate the value of evolution‐guided functional assays to interpret human genetic variation.

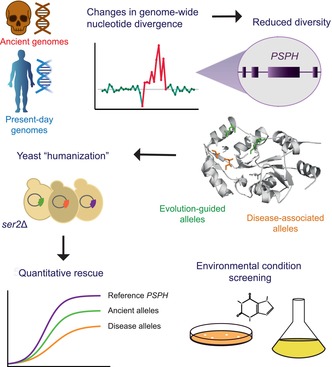

**Abbreviations**
ANOVA: analysis of variance
PSPH: human phosphoserine phosphatase
*R*
: normalized rescue coefficient
*SER2*
: yeast phosphoserine phosphatase gene
WT: Wild type
Δ*π*
: delta nucleotide diversity

L‐serine supports conserved cellular processes including protein synthesis, nitrogen and amino acid metabolism, and the production of essential membrane lipids such as phosphatidylserine, phosphatidylethanolamine, and sphingolipids [1, 2, 3]. In humans, variants in *PSPH*, which encodes phosphoserine phosphatase, are linked to L‐serine deficiency disorders and related neurodevelopmental phenotypes [4, 5, 6]. Functional studies have shown that *PSPH* substitutions such as D32N and M52T can produce a range of effects, from modest or context‐dependent changes to marked reductions in enzyme activity, protein stability and interactions, or cellular phenotypes [4, 7, 8, 9]. Most recently, Sacchi *et al*. [10] reported that the R27S/D32G haplotype, detected in Alzheimer's disease hippocampal samples, reduces catalytic efficiency but does not measurably reduce L‐serine production under heterozygous conditions, while altering subcellular localization. Conversely, *PSPH* overexpression is associated with proliferative and metabolic phenotypes in cancer [11, 12], highlighting the sensitivity of this pathway to changes in *PSPH* dosage, activity, and cellular context.

Given the central metabolic role of *PSPH* and its association with human disease, understanding how genetic variation has shaped its function remains an important challenge. Genome‐wide population methods can detect loci shaped by natural selection in humans and other species (reviewed in [13, 14]), while large‐scale sequencing in clinical and population cohorts has uncovered millions of common and rare human variants [15, 16, 17, 18, 19]. The rapid expansion of ancient DNA datasets adds a temporal dimension to variant interpretation by enabling direct inference of past selection through allele frequency changes over time [20, 21, 22, 23]. Despite these advances, relatively few ancient or archaic alleles have been functionally characterized, with most studies focusing on introgressed regions [24, 25, 26, 27]. Integrating evolutionary signals from ancient and present‐day genomes with scalable functional assays is therefore important for interpreting the biological consequences of human genetic variation.

High‐throughput platforms such as deep mutational scanning, CRISPR perturbation screens, and multiplex variant‐effect assays have expanded our ability to characterize human genetic variation, but they typically measure discrete molecular properties rather than integrated organismal consequences *in vivo* [28, 29, 30, 31]. Humanized animal models provide physiological readouts but are laborious, low throughput, and difficult to scale for rare or ancient alleles [32, 33, 34]. Heterologous complementation in unicellular systems offers a scalable alternative by directly testing whether variants support conserved cellular functions. The phosphorylated serine biosynthesis pathway is conserved from yeast to humans, and *Saccharomyces cerevisiae SER2* encodes the yeast ortholog of human *PSPH* [35]. Yeast *ser2*Δ mutants show severe growth defects in serine‐free media and conditional auxotrophy under glucose limitation, making this system suitable for testing whether human *PSPH* variants can support a conserved cellular function. In *S. cerevisiae*, systematic humanization studies show that many essential genes can be replaced by human orthologs, enabling quantitative complementation assays [36, 37, 38]. Yeast systems can also resolve subtle functional differences among human allelic variants [39, 40, 41].

In this work, we examined whether *PSPH* variants identified in ancient human genomes differ functionally from modern and disease‐associated alleles. Population‐genomic comparisons identified two *PSPH* exons with elevated differences in nucleotide diversity between ancient and present‐day humans, guiding the prioritization of candidate variants. Using yeast *ser*2Δ complementation, we tested R27S and Q83H from this analysis, together with the modern *PSPH* allele and two disease‐associated comparator alleles, D32N and A35T. Quantitative assays across diverse environments revealed reproducible allele‐specific functional differences, with disease‐associated variants showing the strongest impairment and ancient‐genome‐prioritized variants showing more moderate or context‐dependent effects. These findings demonstrate functional divergence among *PSPH* alleles and provide a framework linking evolutionary variant prioritization with scalable heterologous assays.

## Materials and methods

### Ancient genome processing and diversity analyses

Eight ancient human genomes [42, 43, 44, 45, 46, 47, 48], high‐coverage Neanderthal [49] and Denisovan [50] genomes, and 2504 present‐day human genomes from the 1000 Genomes Project Phase 3 call set [15] were analyzed (Table 1). Ancient‐genome reads were mapped to the GRCh38 reference genome and filtered to reduce artifacts associated with low‐quality mapping, sequencing error, and post‐mortem DNA damage. Only reads with mapping quality ≥ 30 were retained, and candidate SNPs were required to have a Phred‐scaled variant quality score ≥ 20. These filters were applied before estimating nucleotide diversity and before prioritizing coding variants for functional analysis. Nucleotide diversity (*π*) was used as the primary summary statistic because it estimates the average number of pairwise nucleotide differences per site within each dataset, providing an allele frequency‐based measure of local sequence variation. This made *π* appropriate for comparing relative diversity across fixed genomic windows between datasets, while avoiding reliance solely on the raw number of segregating sites. Nucleotide diversity (*π*) was estimated separately for the ancient and modern datasets in non‐overlapping 100‐bp windows using Massive Tajima [51]. Differences in nucleotide diversity between datasets were calculated per window as Δ*π*. This analysis was first performed genome‐wide to define the distribution of Δ*π* values and percentile ranks. We then extracted and visualized the *PSPH* region, including the 40 554‐bp *PSPH* locus and ± 100 kb flanking sequence on chromosome 7, for focused analysis.

**Table 1:** Samples, references, and datasets used to estimate the nucleotide diversity of *PSPH*.

Genome/dataset	Location	Sample age (BP)	Lineage context	References/database	Notes
MA‐1	Siberia, Russia	~24 000	Ancient North Eurasian	[45]	Bam file download
Anzick‐1	Montana, U.S.A.	~12 600	Early Native American	[43]	
Kennewick	Washington, U.S.A.	~8500	Early Native American	[46]	
Loschbour	Müllerthal region, Luxembourg	~8000	Western Hunter‐Gatherer	[48]	
Saqqaq	Greenland	~4000	Arctic Hunter‐Gatherer	[42]	
Bichon	Neuchâtel, Switzerland	~13 500	Western Hunter‐Gatherer	[47]	FASTq file download
Kotias	Western Georgia	~9700	Caucasus Hunter‐Gatherer	[47]	
La Braña‐1	León, Spain	~7000	Western Hunter‐Gatherer	[44]	
Denisovan	Denisova Cave, Siberia, Russia	~50 000–100 000	Archaic hominin (Denisovan)	[50]	High‐coverage archaic genomes
Neandertal	Altai Mountains, Siberia, Russia	~50 000	Archaic hominin (Neanderthal)	[49]	
1000 Genomes (Phase 3)	Global	Present day	Modern human populations	[15]	SNP dataset, GRCh38

Because the modern dataset includes substantially more genomes than the ancient dataset (eight genomes), the analysis focused on windows in which nucleotide diversity was higher in ancient genomes than in the 1000 Genomes Phase 3 dataset analyzed here. This filtering strategy was used to avoid prioritizing windows simply because the larger modern panel has greater power to detect rare variants. Diversity differences identified in this way were used to prioritize candidate *PSPH* regions and variants for functional follow‐up; they were not interpreted on their own as evidence for selection, because similar patterns can also arise from demographic history, drift, sampling differences, or other population‐genetic processes.

Coding variants within candidate windows were annotated using Ensembl VEP, and nonsynonymous substitutions present in at least two ancient individuals and absent in modern humans were retained for functional analysis (Data S1). To assess structural conservation between the human PSPH (Uniprot ID: P78330) and the yeast Ser2 (Uniprot ID: P42941) enzymes, the protein structures were superimposed using the “Matchmaker” tool in ChimeraX [52].

### Strains, plasmids, media, and transformation

*S. cerevisiae* BY4741 (MATa *his3*Δ1 *leu2*Δ0 *ura3*Δ0 *met15*Δ0) was used for all transformations. *SER2* was deleted by PCR‐mediated homologous recombination using the LiAc/SS carrier DNA/PEG method. The *kanMX* cassette was amplified from pUG6 and transformants were selected on YPD containing G418 (200 μg·mL^−1^). A *his3*Δ strain generated in parallel served as a wild‐type neutral deletion control (WT). Oligonucleotides for gene disruption and confirmation PCR were obtained from the Yeast Deletion Project. Human *PSPH* (OHu18314C) was amplified from pESC‐URA (GenScript) and yeast *SER2* from S288c genomic DNA. Inserts were cloned into pCM189 [53] using BamHI/PstI sites, ligated with T4 DNA ligase and transformed into *Escherichia coli* DH5α. Constructs were verified by sequencing (Table 2).

**Table 2:** Oligonucleotides used in this study.

Gene	Name	Sequence	Purpose	Notes
*PSPH*	PSPH_27_Fwd	CGGTCATCAGtGAAGAAGGAATC	Site‐directed mutagenesis	R27S substitution
	PSPH_27_Rev	TGCTGTCAACATCAAAAC		
	PSPH_83_Fwd	TAGCAGAGCAcCCCCCACACC	Site‐directed mutagenesis	Q83H substitution
	PSPH_83_Rev	TGAGTCTCTGCACCTGCTC		
	PSPH_32_Fwd	AGAAGGAATCaATGAGCTAGCC	Site‐directed mutagenesis	D32N substitution
	PSPH_32_REV	TCTCTGATGACCGTGCTG		
	PSPH_35_Fwd	CGATGAGCTAaCCAAAATCTG	Site‐directed mutagenesis	A35T substitution
	PSPH_35_Rev	ATTCCTTCTTCTCTGATGAC		
	PSPH_Fwd	ctagtaggatccATGGTCTCCCACTCAGAGCTG	Cloning *PSPH* into pCM189 vector	Restriction site BamHI/PstI
	PSPH_Rev	caagcgctgcagTTATTCTTCCAGTTCTCCCAG		
*SER2*	SER2_UPstream45	CAAGTCCAATAGCATAGACATTAAGCACGACAGCTGTAAAAAATGccagctgaagcttcgtacgc	Knockout strain generation	Deletion strain *∆ser2*
	SER2_DNstream45	TTTAAAAAGAAACTCTATTACATCTATCTATCATTATTTTCTTCAtcgatgaattcgagctcgtt		
	SER2_Confi_A	ACCCTTTTCACCGGAACTAATATAC	Knockout confirmation	*∆ser2* deletion confirmation
	SER2_Confi_B	CAACAAGTTTGCCTTACAAAAATCT		
	SER2_Fwd	cgagtaggatccATGTCAAAGTTTGTTATCACC	Cloning into pCM189 vector	Restriction site BamHI/PstI
	SER2_Rev	ccagcgctgcagTCATTGTCTATTGTATATTTC		
*HIS3*	HIS3_F1	ACTAAAAAATGAGCAGGCAAGATAAACGAAGGCAAAGATGCCAGCTGAAGCTTCGTACGC	Knockout strain generation	Reference *∆his3* deletion strain
	HIS3_R2	ATATATCGTATGCTGCAGCTTTAAATAATCGGTGTCACTATCGATGAATTCGAGCTCGTT		
	HIS3_A	TGACGACTTTTTCTTAATTCTCGTT	Knockout confirmation	*∆ser2* deletion confirmation
	HIS3_B	CTATGTGTAAGTCACCAATGCACTC		
	pcM189_FWD	cctgtaggtcaggttgctttc	Insert confirmation and sequencing	Candidate gene verification
	pcM189_REV	TCGGTTAGAGCGGATGTG		
	KanB	ctgcagcgaggagccgtaat	Universal reverse knockout confirmation primer	Primer at *kanMX* resistance marker

Yeast knockout strains were transformed with pCM189 constructs and selected on SC medium lacking ammonium and uracil, supplemented with G418 (200 μg·mL^−1^) and monosodium glutamate (1 g·L^−1^). The amino acid supplement mix contains no L‐serine. Transformation mixtures were first plated on non‐selective medium and then replica‐plated onto selective medium. Colonies growing on selective medium were picked as independent transformant colonies.

### Site‐directed mutagenesis of human *PSPH*

Missense variants of human *PSPH* were generated from pCM189‐*PSPH* by site‐directed mutagenesis using 25‐bp mutagenic primers (Table 2). PCR amplification was performed with Phusion HotStart II polymerase (Thermo), followed by phosphorylation with T4 polynucleotide kinase and ligation with T4 DNA ligase. Template plasmids were removed by DpnI digestion. Products were transformed into *Escherichia coli* and selected on LB agar with ampicillin (100 μg·mL^−1^). Plasmids were sequence‐verified, yielding pCM189‐*psph* ^R27S^, ‐*psph* ^D32N^, ‐*psph* ^A35T^, and ‐*psph* ^Q83H^.

### Yeast dilution spot assays

Dilution spot assays were used to assess growth defects of *ser2*Δ cells on SC medium lacking serine and complementation by human *PSPH*. Pre‐cultures were grown in SC medium for 48 h at 30 °C and normalized to OD_600_ = 1. Five 10‐fold serial dilutions were prepared and 3 μL of each dilution was spotted onto SC agar plates. Plates were incubated at 30 °C and imaged after 72 h.

### Growth assays under environmental perturbations and data analysis

Functional effects of human *PSPH* missense variants were evaluated across multiple environmental conditions. Cells were grown in SC or SC supplemented with chemical stressors or alternative carbon sources (Table 3). For each construct and condition, five independent verified transformant colonies were analyzed as biological replicates, each measured in three to six technical replicates. All technical replicates were averaged before statistical analysis, which considered only the independent transformant colony replicates (Data S2).

**Table 3:** Environmental conditions tested for yeast growth assays.

Abbreviation	Condition	Cellular pathway targeted
SCD^a^	Dextrose 2%	Reference condition
SC–G	Galactose 2%	Carbon source replacement
SC–S	Sucrose 2%	Carbon source replacement
SC–R	Raffinose 2%	Carbon source replacement
AmB	Amphotericin B 1.6 μg·mL^−1^	Membrane stress antifungal, ergosterol binding
BEN	Benomyl 10 μm	Microtubule stress
CaCl_2_	CaCl_2_ 0.12 mm	Ionic and Ca^2+^ signaling stress
CAF	Caffeine 0.2%	TOR inhibition and nutrient signaling stress
CuSO_4_–1	CuSO_4_ 0.6 mm	Copper toxicity and oxidative stress
CuSO_4_–2	CuSO_4_ 0.5 mm	Copper toxicity and oxidative stress
CuSO_4_–3	CuSO_4_ 0.3 mm	Copper toxicity and oxidative stress
ETA–1	Ethanolamine 10 mm	Membrane lipid metabolism stress
ETA–2	Ethanolamine 2 mm	Membrane lipid metabolism stress
ETA–3	Ethanolamine 5 mm	Membrane lipid metabolism stress
ETA–4	Ethanolamine 80 mm	Membrane lipid metabolism stress
ETH	Ethanol 3%	Membrane and solvent stress
HPD	Hesperidin 100 μm	Oxidative stress and antioxidant response
HU	Hydroxyurea 80 mm	DNA replication stress
KCl–1	KCl 0.5 M	Osmotic and ionic stress
KCl–2	KCl 1 M	Osmotic and ionic stress
LA	Lactic acid 1.5%	Organic acid stress
LCA–1	Lithocholic acid 0.1 mm	Membrane and bile acid stress
LCA–2	Lithocholic acid 0.78 mm	Membrane and bile acid stress
MEN	Menadione 2 mm	Oxidative stress (ROS)
MTF	Metformin 50 mm	Mitochondrial respiration stress
MMS	Methyl methanesulfonate 0.01%	DNA damage
MnSO_4_–1	MnSO_4_ 0.1 mm	Metal ion stress
MnSO_4_–2	MnSO_4_ 1 mm	Metal ion stress
PQ	Paraquat 2 mm	Oxidative stress (superoxide)
H_2_O_2_–1	Hydrogen peroxide 0.5 mm	Oxidative stress (ROS)
H_2_O_2_–2	Hydrogen peroxide 1 mm	Oxidative stress (ROS)
SDS	Sodium dodecyl sulfate 0.01%	Membrane stress
NaAc–1	Sodium acetate 0.5%	Weak acid stress
NaAc–2	Sodium acetate 1%	Weak acid stress
NaCl	NaCl 0.4 M	Osmotic and ionic stress

^a^ SCD was synthetic complete medium without uracil supplemented with 2% glucose, 0.1% monosodium glutamate used as the nitrogen source, and 200 μg·L^−1^ geneticin for plasmid selection. The amino acid supplement mix contains no L‐serine.

Pre‐cultures were grown in 96‐well plates containing SC (150 μL) for 48 h at 30 °C. Saturated cultures were resuspended by mixing, and 10 μL of each culture was transferred to 200 μL of fresh assay medium in 96‐well plates. Blank wells containing the corresponding assay medium without cells were included for each condition and used for background correction of OD_600_ values. Growth assays were performed using a Tecan EVO 200 robotic station integrated with an Infinite M1000 plate reader. Plates were incubated at 30 °C and 70% relative humidity without continuous shaking; immediately before each reading, plates were shaken for 30 s at 1000 rpm. OD_600_ was measured every hour for 30–40 h. Growth parameters, including relative growth rate, were calculated from blank‐corrected OD_600_ growth curves after averaging three to six measurements for each biological replicate.

Growth curves were analyzed using MATLAB (R2021a) and R (v4.3.0); visualizations were generated with *ggplot2* (v3.4.2). Growth rates (*G*) were calculated from doubling times derived from the mean of the second and third maximal slopes of ln‐transformed growth curves. Mean *G* values were computed from biological replicates for each strain and condition. Phenotypic rescue was quantified using the metric *R*, calculated within each condition as *R* = (*G*_*pX*_–*G*_*p0*_)/(*G*_*p1*_–*G*_*p0*_), where *G*_*pX*_ is the growth rate of the strain carrying a given construct, *G*_*p0*_ is the mean growth rate of *ser2*Δ cells carrying empty pCM189 vector, and *G*_*p1*_ is the mean growth rate of *ser2*Δ cells expressing yeast *SER2* from pCM189. Under this definition, *R* = 0 indicates no rescue, *R* = 1 indicates full rescue equivalent to yeast *SER2*, and intermediate values indicate partial complementation. Statistical differences between constructs were assessed within each condition by one‐way ANOVA followed by Tukey's HSD test (Data S2). For cumulative distributions of *R* across environmental conditions, allele‐level differences were assessed using pairwise two‐sample Kolmogorov–Smirnov tests followed by Benjamini–Hochberg correction.

## Results

### Genome‐wide nucleotide diversity analysis prioritizes *PSPH* candidate variants in ancient and present‐day human datasets

**Fig. 1:**
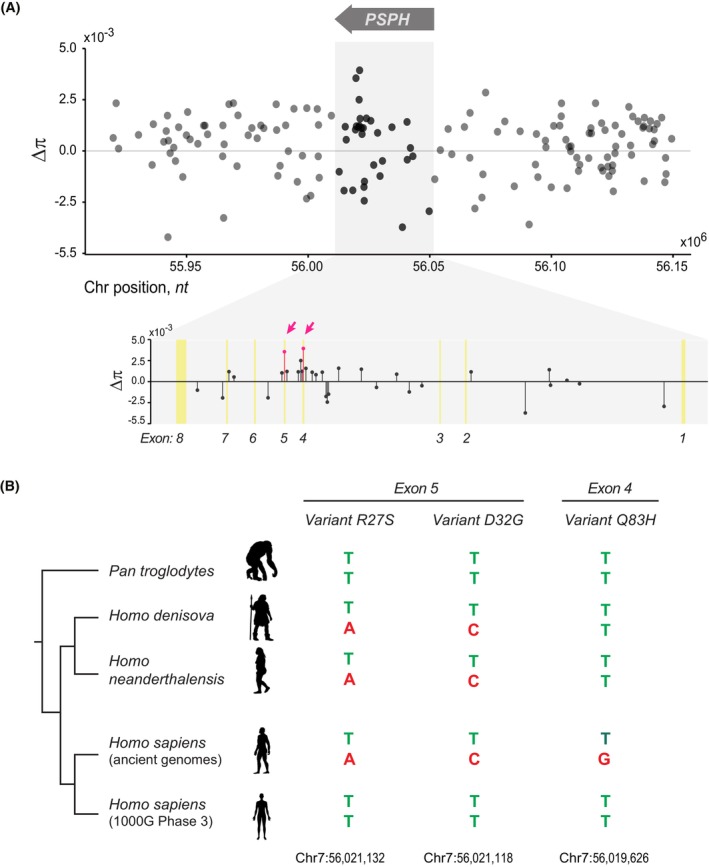
Nucleotide diversity at the human phosphoserine phosphatase (*PSPH*) in ancient and modern genomes. (A) Differences in nucleotide diversity (*π*) between eight ancient genomes and present‐day humans in the 1000 Genomes Phase 3 call set across the *PSPH* genomic region (± 100 kilobases). Each point represents the difference in nucleotide diversity (Δ*π*) between datasets, calculated in non‐overlapping 100‐bp windows using Massive Tajima (Flores‐Ponce et al., 2017). The gray box marks the *PSPH* locus; values within this region are highlighted as dark data points. The lower panel shows Δ*π* values restricted to the *PSPH* locus; coding regions (exons) are indicated in yellow and high‐diversity windows within exons are highlighted in pink. (B) Nucleotide states at three coding positions identified in high‐diversity windows. Sites were selected when supported by at least two ancient genomes and producing nonsynonymous substitutions. Alleles are shown across hominid genomes with chimpanzee used as outgroup. The present‐day human comparison refers specifically to the filtered 1000 Genomes Phase 3 call set analyzed in this study.

To investigate evolutionary patterns in the serine biosynthesis pathway, we first compared nucleotide diversity observed in eight ancient human genomes with nucleotide diversity in the 1000 Genomes Phase 3 present‐day human dataset (Table 1). We used Δ*π*, a metric that captures differences in nucleotide diversity between groups [15]. We then focused on *PSPH* because two coding windows within this gene showed elevated Δ*π* values relative to the genome‐wide distribution. We examined the *PSPH* locus (40 554 bp) and scanned 100‐bp windows across a ± 100 kb interval flanking the gene on chromosome 7, surveying ~241 kb in total using Massive Tajima [51]. Using this approach, we identified two exonic windows within *PSPH* showing elevated Δ*π* values, indicating localized diversity shifts between ancient genomes and the 1000 Genomes Phase 3 dataset analyzed here (Fig. 1A). Exon 4 exhibited a Δ*π* peak of 3.94 × 10^−3^ and exon 5 a peak of 3.55 × 10^−3^, ranking within the top 1.42% and 1.86% of genome‐wide windows, respectively. These regions contained 14 variants (Data S1), including five in exon 4 (two nonsynonymous) and nine in exon 5 (six nonsynonymous).

For functional prioritization, we focused on variants that produced nonsynonymous substitutions and were supported by at least two ancient human genomes. We prioritized three nonsynonymous variants (R27S, D32G, and Q83H) supported by at least two ancient genomes and high sequencing quality metrics (Fig. 1B). Notably, R27S and D32G alleles were also present in Neanderthal and Denisovan genomes. Because the present‐day comparison was based on the filtered 1000 Genomes Phase 3 call set, we evaluated whether the absence of R27S/rs74445297 and D32G/rs78599516 reflected limited representation of the *PSPH* locus in that dataset. Neither variant was detected as polymorphic at the analyzed positions in this filtered 1000 Genomes Phase 3 call set, although nearby *PSPH* polymorphisms were represented across the full sample set. Thus, their absence reflects the specific 1000 Genomes Phase 3 call set used for our screen. Consistent with Sacchi *et al*. [10], both variants are reported in broader contemporary resources such as gnomAD v4.1.0, supporting their description as older *PSPH* polymorphisms retained at variable frequencies in present‐day populations. Together, these variants cluster within exons overlapping pronounced Δ*π* peaks provided a focused set of candidate *PSPH* alleles for functional follow‐up.

Functional annotation using *SIFT*, *PolyPhen‐2*, and *REVEL* predicted neutral effects for the ancient Q83H and R27S variants. Q83H was classified as tolerated/benign/neutral (scores: 1.0, 0.00, 0.227), and R27S showed similar predictions (1.0, 0.007, 0.461). These concordant results suggest that both substitutions are unlikely to be deleterious. In contrast, D32G was predicted to be deleterious (*SIFT* 0.02), probably damaging (*PolyPhen‐2* 0.941) and with a possible functional effect (*REVEL* 0.692), consistent with prior evidence linking residue 32 to *PSPH*‐associated disease [4, 54]. Together, these results identified *PSPH* coding variants in exons with elevated diversity differences between the ancient and modern datasets analyzed here and provided a focused set of candidate alleles for functional testing.

### Yeast complementation reveals allele‐specific functional differences among human *PSPH* variants

**Fig. 2:**
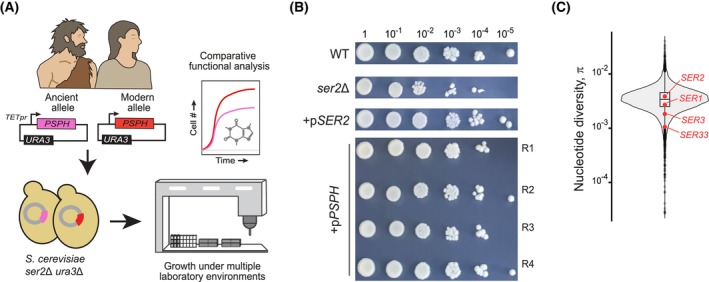
Heterologous complementation of yeast *SER2* by human phosphoserine phosphatase (*PSPH*). (A) Experimental design for testing complementation of a yeast *ser2*Δ mutant with human *PSPH*. (B) Dilution spot assays comparing growth of wild‐type (WT), *ser2*Δ, and *ser2*Δ strains complemented with yeast *SER2* or human *PSPH*. Genes were expressed from the centromeric plasmid pCM189 under control of the *tetO7* promoter. Independent verified transformant colonies were replica‐plated before the assay, so each spot series represents an independent biological replicate. Saturated cultures were normalized at OD_600_ = 1.0 before serial dilution and spotting. (C) Violin plots showing the distribution of coding‐sequence nucleotide diversity (*π*) across all genes in the 1 011 *S. cerevisiae* genomes panel. Genes coding for enzymes in the main serine biosynthesis pathway are highlighted in red. This analysis places *SER2* diversity in the internal context of *S. cerevisiae* coding genes and helps evaluate whether it is an unusually low‐diversity gene within the yeast complementation system.

The *PSPH* variants R27S, D32G, and Q83H show a striking evolutionary pattern whereby alternative alleles occur in multiple ancient and archaic human genomes while displaying marked differences between ancient and present‐day human datasets despite some of them being predicted neutral by sequence‐based tools. Among these variants, R27S and Q83H were predicted to be functionally neutral by sequence‐based tools, whereas D32G was predicted to have a functional effect. This combination of evolutionary prioritization and heterogeneous computational predictions motivated experimental validation. To test functional conservation across species, we examined whether human *PSPH* complements deletion of its ortholog in *S. cerevisiae*. Although human and yeast *PSPH* share only ~21% sequence identity, both catalyze the final step of the conserved L‐serine biosynthesis pathway. We therefore developed a quantitative heterologous complementation assay measuring growth dynamics rather than endpoint rescue (Fig. 2A). This framework enables sensitive detection of graded functional differences and direct comparison among modern, ancient, and disease‐associated PSPH variants across diverse environmental conditions.

To first test complementation qualitatively, we performed drop‐spot assays using independent verified *ser2*Δ transformant colonies carrying the indicated plasmids. The *ser2*Δ mutant carrying empty vector showed a pronounced growth defect under conditions requiring endogenous serine biosynthesis (Fig. 2B, *top*). Expression of human *PSPH* from a *Tet*‐regulated centromeric vector (pCM189) restored growth across independent transformants, demonstrating functional complementation of yeast *SER2* (Fig. 2B, *bottom*). These results indicate conserved phosphoserine phosphatase function across eukaryotes. To provide context for the yeast gene used in the complementation assay, we examined coding‐sequence diversity of *SER2* in the 1011‐genomes *S. cerevisiae* panel [55]. *SER2* showed nucleotide diversity above the genome‐wide median and the highest *π* among the main serine biosynthesis genes (Fig. 2C), indicating that the yeast ortholog does not appear unusually constrained relative to other genes in the pathway within *S. cerevisiae*. This result indicates that *SER2* is not an extreme low‐diversity outlier within *S. cerevisiae* and supports its use as the endogenous yeast reference in the complementation assay.

**Fig. 3:**
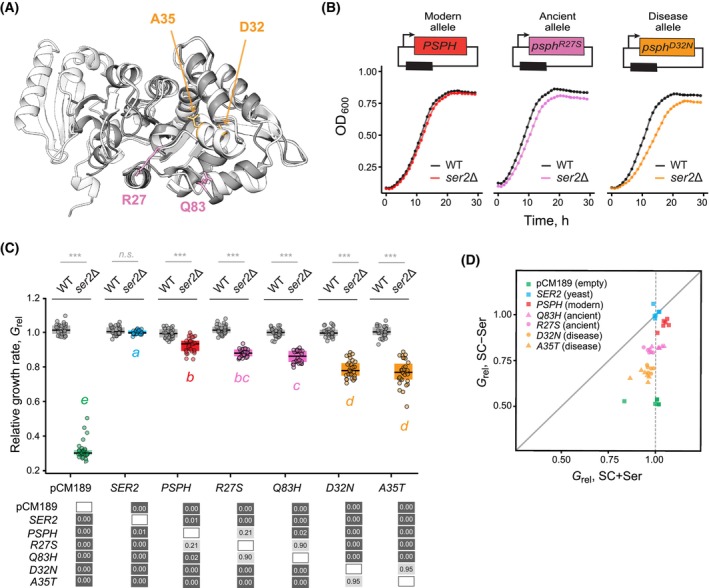
Human phosphoserine phosphatase (*PSPH*) variants show allele‐specific complementation of yeast *ser2*Δ. (A) Structural alignment of human PSPH (gray; Protein Data Bank [PDB]: 6HYJ) and the AlphaFold‐predicted *S. cerevisiae* Ser2 structure (white) generated with ChimeraX. Residues corresponding to ancient‐genome‐prioritized *PSPH* variants tested in yeast are highlighted in pink, and disease‐associated comparator residues are highlighted in orange. (B) Growth curves of wild‐type (WT; *his3*Δ, black) and *ser2*Δ strains carrying plasmids expressing the indicated genes or variants. (C) Relative growth rates of each strain normalized to WT (*his3*Δ + pCM189). Gray shading indicates WT, whereas colored boxes represent *ser2*Δ strains, each expressing the indicated genes or variants. Boxplots show the median and interquartile range of all individual measurements, which are overlaid as data points. Data were obtained from five independent transformant colonies, each measured six times; technical measurements were averaged by colony before statistical analysis (*n* = 5). Direct WT versus *ser2*Δ comparisons are shown above the plot (***P < 0.001; ^n.s.^ *P* ≥ 0.05). Growth differences within the WT and *ser2*Δ complementation series were analyzed separately by ANOVA with Tukey's multiple comparison test. Adjusted pairwise *P*‐values are shown below the plot for the *ser2*Δ complementation series, with dark boxes indicating significant comparisons. Compact letter displays shown below each boxplot indicate Tukey groupings; groups sharing a letter are not significantly different. (D) L‐serine supplementation assay showing relative growth rates (*G*) of the indicated strains in medium with or without L‐serine added (375 mg·L^−1^). Each data point corresponds to one independent transformant colony measured once (*n* = 5). The dashed vertical line indicates full growth restoration in medium supplemented with L‐serine.

After confirming qualitative complementation, we used the haploid *ser2*Δ background to compare allele‐specific complementation capacity under the same cellular context because each *PSPH* allele provides the sole complementing phosphoserine phosphatase activity. After confirming complementation, we tested whether *PSPH* allelic variation affected complementation strength *in vivo*. The variant panel included two classes of substitutions. First, we selected R27S and Q83H from the population‐genomic analysis because they were nonsynonymous variants supported by multiple ancient genomes and predicted to be neutral by sequence‐based tools. The same analysis also identified D32G, including in Neanderthal and Denisovan genomes; however, D32G was not included in the yeast complementation panel. Second, we included two disease‐associated comparator alleles, D32N and A35T, to provide internal references for impaired PSPH function. D32N affects the same residue as D32G and therefore provides a disease‐associated comparison at this position [4, 5, 54]. Structural alignment of human PSPH with predicted *S. cerevisiae* Ser2 showed strong overlap in the N‐terminal region containing these residues, supporting yeast complementation as a readout of conserved phosphoserine phosphatase function (Fig. 3A).

We quantified complementation by measuring liquid growth kinetics in WT and ser2Δ backgrounds carrying empty vector, yeast *SER2*, modern human *PSPH*, the ancient‐genome‐prioritized variants R27S and Q83H, or the disease‐associated comparator variants D32N and A35T. For each construct, five independent verified transformant colonies were selected and used as biological replicates, with each biological replicate assayed in six technical replicates in 96‐well growth assays. Representative growth curves showed reproducible differences in growth rate and carrying capacity among alleles relative to controls (Fig. 3B). In the *ser2*Δ background strains, one‐way ANOVA identified highly significant differences among plasmid constructs (*P* < 10^−23^), and Tukey's HSD *post hoc* test resolved distinct complementation groups. Yeast *SER2* produced the strongest rescue and was indistinguishable from WT, whereas the modern human *PSPH* reference allele also provided strong rescue, but at a significantly lower level than the yeast ortholog (Fig. 3C). Among the *PSPH* missense variants, the disease‐associated alleles showed the weakest rescue, whereas the ancient‐genome‐prioritized variants showed milder, intermediate effects. The two disease‐associated alleles were indistinguishable from each other, while the ancient‐genome‐prioritized variants occupied intermediate positions in the Tukey HSD grouping. Q83H differed from the modern PSPH reference allele in this single‐condition assay, whereas R27S showed a milder effect; R27S and Q83H also differed modestly from each other in the pairwise Tukey comparison (*p*_adj_ = 0.03; Fig. 3C). To confirm that these growth differences reflected serine biosynthesis capacity, we supplemented cultures with exogenous L‐serine (375 mg·L^−1^). Serine supplementation generally restored growth of the *ser2*Δ mutant and reduced growth differences among *PSPH* alleles, indicating that the observed phenotypes primarily reflect limitation of serine biosynthesis rather than unrelated effects of *SER2* deletion or heterologous *PSPH* expression (Fig. 3D). Together, these results show that human *PSPH* can complement yeast *SER2* loss and that *PSPH* missense variants differ quantitatively in their capacity to support this conserved cellular function.

### Environmental profiling reveals context‐dependent modulation of *PSPH* allele effects

**Fig. 4:**
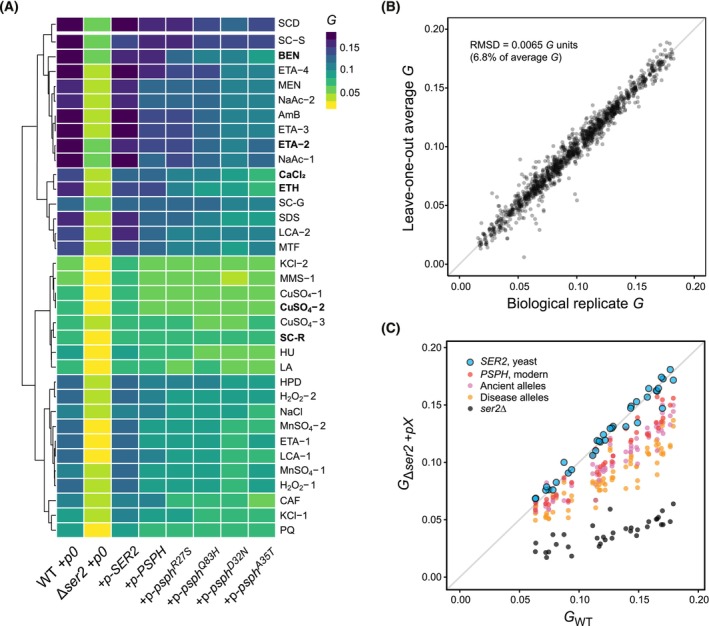
Functional complementation of yeast *ser2*Δ by human phosphoserine phosphatase (*PSPH*) variants across environmental conditions. (A) Heat map of relative growth rates of *ser2*Δ strains complemented with human *PSPH* and four missense variants across 35 environmental conditions (abbreviations in Table 2). Yeast *SER2* and the empty vector (p*0*) were included as positive and negative controls in wild‐type (WT) and *ser2*Δ backgrounds. Conditions highlighted in bold are those identified as showing significant condition‐dependent changes in allele effects in the analysis presented in Fig. 5. Values represent means from five independent transformant colonies; technical triplicates were averaged before plotting and statistical analysis. (B) Scatter plot showing replicate agreement between each biological replicate growth rate estimate and the leave‐one‐out mean of the remaining four biological replicates for the same strain/condition. Technical triplicates were averaged before calculating biological replicate values. The gray line indicates *y* = *x*. Reproducibility was summarized as the root mean square deviation (RMSD) from the leave‐one‐out mean; the low RMSD value indicates a high agreement among biological replicates. (C) Relationship between growth rates of *ser2*Δ strains expressing different alleles and those of WT + p*0* across environmental conditions. Colors denote allele classes: yeast *SER2* (blue), modern human *PSPH* (red), ancient variants (pink), disease‐associated variants (orange), and empty vector (black).

Our results showed that heterologous complementation in yeast is sufficiently sensitive to detect subtle functional differences among *PSPH* variants. To determine whether environmental context modulates these differences, we measured complementation across 35 conditions, including alternative carbon sources, nutrient‐related perturbations, ionic and metal stresses, ethanol exposure, DNA replication stress, cytoskeletal perturbation, and other challenges to cellular homeostasis (Table 3). These conditions were chosen to sample a wide range of cellular states and stress responses in which genetic perturbations often produce condition‐dependent phenotypes in yeast [56, 57]. This strategy is particularly relevant for metabolic enzymes because their effects can depend on nutrient availability, stress physiology, and broader cellular network state. For PSPH, which catalyzes the final step of L‐serine biosynthesis, such contexts may influence the demand for serine‐derived processes, including protein synthesis, one‐carbon metabolism, and lipid metabolism. We therefore used the environmental panel to test whether *PSPH* allelic differences were robust across conditions or instead revealed genotype‐by‐environment effects. Growth profiles across conditions for *ser2*Δ strains carrying empty vector, yeast *SER2*, modern *PSPH*, or *PSPH* allelic variants are summarized in Fig. 4A and reported in Data S2. Using a leave‐one‐out approach, we observed high consistency among the five independent transformant‐colony replicates, with a global normalized RMSD of 6.8% across the assay (Fig. 4B). In addition, the strong concordance between WT cells carrying empty plasmid and *ser2*Δ cells complemented with *SER2* (*r* = 0.985, *P* < 10^−15^; Pearson correlation) indicates that centromeric‐plasmid complementation preserved growth patterns across the tested environments (Fig. 4C). As expected, the *ser2*Δ strain carrying empty vector showed the strongest growth impairment, whereas *PSPH* variants conferred partial but reproducible rescue.

**Fig. 5:**
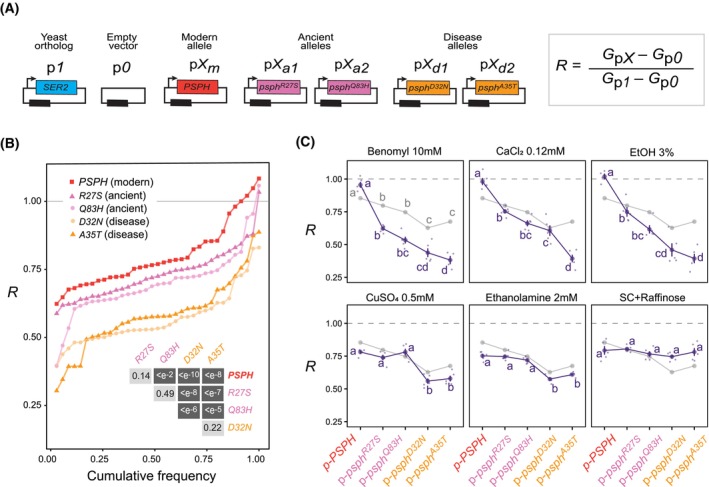
Rescue coefficient analysis reveals context‐dependent rescue by human phosphoserine phosphatase (*PSPH*) variants. (A) Plasmid constructs used in the assay (top) and definition of the normalized rescue coefficient (*R*) used to quantify complementation (bottom), where *G*_pX_ is the growth rate of the tested allele, *G*_p1_ is the growth rate of the yeast *SER2* reference, and *G*_p0_ is the growth rate of the empty vector control. (B) Cumulative distribution of *R* values for modern *PSPH* (red) and its variants across environmental conditions. Ancient variants are shown in pink and disease‐associated variants in orange. *Inset*, Benjamini–Hochberg adjusted *P*‐values from pairwise Kolmogorov–Smirnov tests; dark cells indicate significant differences. (C) Normalized *R* of strains expressing different *PSPH* variants across environmental conditions (purple). Large circles indicate means and small circles individual biological replicates; vertical bars denote SEM. The reference condition (synthetic complete medium without uracil supplemented with 2% glucose, 0.1% monosodium glutamate used as the nitrogen source, and 200 μg·L^−1^ geneticin for plasmid selection. The amino acid supplement mix contains no L‐serine; SCD) is shown in gray. Lowercase letters indicate groups not significantly different within each condition (ANOVA with Tukey's *post hoc* test; Data S2).

To quantify complementation across conditions, we defined a rescue metric (*R*) based on specific growth rates scaled between mutant (no complementation) and wild‐type (full complementation) controls (Fig. 5A). This normalization expresses variant performance as a continuous measure and enables condition‐independent comparison of complementation. Cumulative distributions of *R* across 35 conditions revealed a consistent complementation hierarchy among *PSPH* variants (Fig. 5B). Pairwise Kolmogorov–Smirnov tests with Benjamini–Hochberg correction showed that the modern allele had significantly stronger rescue than the disease‐associated variants and Q83H, but did not differ significantly from R27S across the full distribution of environmental conditions (Fig. 5B, *bottom*). Disease‐associated variants exhibited the weakest complementation and were indistinguishable from each other, yet differed significantly from both ancient alleles. The two ancient variants were modestly but significantly different from each other. Together, these results indicate a consistent pattern across environments, in which the modern *PSPH* allele generally showed stronger complementation, Q83H showed reduced rescue relative to modern *PSPH*, R27S was closer to the modern allele in the across‐condition distribution, and the disease‐associated alleles showed the weakest rescue.

Despite the reproducible pattern of allele‐specific differences, a small subset of environmental stresses altered the magnitude of these differences. In some conditions, the separation between the modern allele and one or more variant alleles increased, widening performance gaps without changing the overall direction of the effects. This pattern was observed under stresses imposed by 10 μm benomyl, 0.12 mm CaCl_2_, and 3% ethanol (Fig. 5C, *top*), which perturb distinct aspects of yeast physiology, including microtubule function, calcium‐dependent stress responses, and membrane homeostasis, respectively. Thus, these conditions may expose *PSPH* allele‐specific differences that are partially buffered under standard growth conditions, although they do not establish a direct mechanistic link between *PSPH* and each stress pathway. Conversely, a limited number of environments alleviated the defects of specific variant classes, including conditions that reduced differences between the modern allele and one or both ancient‐genome‐prioritized variants, such as in 0.5 mm CuSO_4_ and 2 mm ethanolamine, as well as raffinose, a non‐repressing carbon source that alleviated the deficits of disease‐associated alleles (Fig. 5C, *bottom*) (ANOVA followed by Tukey's HSD test, see Data S2). Together, these context‐dependent changes indicate that, while the main allele‐specific trends are broadly reproducible, environmental perturbations can modulate the magnitude of allelic effects and uncover genotype–environment interactions that are not evident in single‐condition assays.

## Discussion

This study reports the functional complementation of the *S. cerevisiae ser2*Δ mutant by human *PSPH*, establishing a cross‐species *in vivo* assay to compare human *PSPH* alleles selected from evolutionary and clinical contexts. Moving beyond binary complementation readouts [36], our complementation approach resolved quantitative differences among *PSPH* variants. Across growth conditions, the modern reference allele generally supported stronger complementation than the tested variants, with the clearest differences observed relative to the disease‐associated comparator alleles D32N and A35T, whereas the ancient‐genome prioritized variants R27S and Q83H showed measurable but more condition‐dependent rescue. Among these, Q83H showed clearer reduction relative to modern *PSPH*, whereas R27S showed a more modest and condition‐dependent effect. Importantly, the two ancient‐genome‐prioritized variants tested here were predicted to be functionally neutral by sequence‐based approaches, indicating that the yeast assay captures subtle *in vivo* effects that are not readily detected by standard computational predictors. Together, these results show that human *PSPH* variation can produce measurable allele‐specific effects in the context of a living eukaryotic cell.

The evolutionary component of our study was used to prioritize variants for functional testing. Because the ancient and present‐day datasets differ substantially in size and sampling structure, the resulting diversity patterns are best interpreted as a signal that may reflect selection, demography, drift or sampling history. Comparisons between ancient and modern genomes identified *PSPH* substitutions whose frequency patterns made them candidates for experimental analysis, and the yeast assay showed that the two tested candidates, R27S and Q83H, differ functionally from the modern reference allele. The variants prioritized here also showed uneven representation across modern datasets and populations: R27S, D32G and Q83H were not observed as polymorphic variants in the 1000 Genomes Phase 3 dataset despite representation of the surrounding *PSPH* region, whereas larger contemporary resources such as gnomAD indicate that they are present in modern humans, particularly in African and African‐American ancestry groups and at lower frequencies in some East Asian populations [18]. This distribution is consistent with older standing variation retained or shifted in frequency across human populations. As illustrated by metabolic loci such as lactase persistence, where changing dietary and ecological contexts reshaped allele‐frequency trajectories [58], such patterns can motivate experimentally testable hypotheses about metabolic function. In *PSPH*, our results raise the possibility that human evolutionary history may harbor functional polymorphisms whose effects are subtle, context‐dependent and unevenly represented across present‐day populations.

Our findings align with prior biochemical and cellular analyses showing that allelic *PSPH* variants can affect enzyme function through multiple mechanisms. Because PSPH catalyzes the final step of L‐serine biosynthesis, it contributes to serine supply for protein synthesis, one‐carbon metabolism, phospholipid production and nervous system serine homeostasis [1, 2, 3]. Previous studies of *PSPH* variants have reported effects on catalytic efficiency, ligand or cofactor response, protein stability, cellular abundance and subcellular localization [4, 5, 8, 9, 10]. This prior work is particularly relevant for the N‐terminal region of PSPH. Recently, Sacchi *et al*. described an R27S/D32G *PSPH* variant detected in Alzheimer's disease brain samples and reported altered cellular behavior, including increased nuclear localization [10]. Together with the presence of R27S and D32G in contemporary resources such as ClinVar and gnomAD [18, 59], these observations highlight an overlap between variants prioritized by ancient–modern diversity comparisons and variants observed in contemporary clinical or population datasets, while emphasizing the need for direct functional and genetic‐context analyses before drawing conclusions about pathogenicity. Our experimental panel included R27S as a single substitution, showing that this variant can produce modest, condition‐dependent changes in yeast complementation and supporting the functional sensitivity of the N‐terminal region of PSPH.

The yeast complementation assay provides a scalable haploid, allele‐specific growth readout in which each *PSPH* variant is tested as the sole complementing phosphoserine phosphatase activity in the *ser2*Δ background. Thus, the assay measures the capacity of individual *PSPH* variants to support a conserved cellular function in yeast, complementing biochemical and human‐cell studies in which heterozygous wild‐type *PSPH* activity may buffer variant effects on L‐serine production. The functional conclusions from this assay are restricted to the variants directly tested, namely R27S and Q83H as evolutionarily prioritized variants and D32N and A35T as disease‐associated comparators. The absence of D32G from the yeast complementation panel is therefore a limitation of the present study. Moreover, the variant panel does not provide a comprehensive *PSPH* variant‐effect map; the relative behavior of untested alleles, including D32G and M52T, will require direct experimental analysis. The prioritized variants are relevant because they occur in temporally stratified human genomes; broader mutational screens will be needed to define the full *PSPH* functional landscape. Reduced complementation in *ser2*Δ yeast may reflect altered catalytic efficiency, protein stability, or interactions within the serine‐biosynthesis network, mechanisms that cannot be distinguished by growth rescue alone. The assay also does not measure human tissue‐specific regulation, disease penetrance, or physiological consequences *in vivo*. These limitations underscore the central interpretation that clinical databases, disease‐associated studies, and ancient genomes can identify *PSPH* substitutions as candidates for functional follow‐up, but each variant and haplotype requires direct experimental assessment.

Our study connects ancient‐genome analysis, clinical variant prioritization, and experimental functional biology in a single workflow. Ancient and modern genome comparisons can help prioritize variants that are not obvious from clinical annotation, present‐day frequency alone, or *in silico* prediction, while yeast complementation can rapidly test whether those variants alter *PSPH*‐dependent growth *in vivo*. Within this framework, temporal population genomics guides the prioritization of functionally informative metabolic variation, including polymorphisms whose effects are subtle, context‐dependent, and unevenly represented across present‐day populations. As ancient genome datasets and clinical variant catalogs continue to expand, integrating evolutionary genomics with scalable functional assays will be essential for identifying variants whose biological significance may not be apparent from modern datasets alone.

## Conflict of interest

The authors declare no conflict of interest.

## Author contributions

MC‐A: conceptualization, investigation, formal analysis, visualization, writing – original draft. DA: investigation, visualization, writing – original draft. MV‐E: conceptualization and formal analysis. EG‐O: formal analysis. CEM‐G: formal analysis. RM: conceptualization, supervision, funding acquisition, writing – original draft. AD: conceptualization, supervision, funding acquisition, writing – original draft. All authors read and critically revised the manuscript.

## Supporting information


**Data S1.** Single‐nucleotide variants in *PSPH* exons identified from genome analysis.

**Data S2.** Growth rate data and statistics of complemented mutants under 35 conditions.

## Data Availability

All processed data are provided in Data S1 and Data S2 accompanying this article as Supporting Information. Raw data will be made available from the corresponding author upon reasonable request.
